# Int-HRL: towards intention-based hierarchical reinforcement learning

**DOI:** 10.1007/s00521-024-10596-2

**Published:** 2024-12-11

**Authors:** Anna Penzkofer, Simon Schaefer, Florian Strohm, Mihai Bâce, Stefan Leutenegger, Andreas Bulling

**Affiliations:** 1 Institute for Visualisation and Interactive Systems, University of Stuttgart, Pfaffenwaldring 5A, 70569 Stuttgart, Germany; 2 Machine Learning for Robotics, Technical University of Munich, Boltzmannstrasse 3, 85748 Munich, Germany; 3 Department of Computer Science, KU Leuven, Andreas Vesaliusstraat 13, box 2600, 3000 Leuven, Belgium

**Keywords:** Hierarchical reinforcement learning, Intention prediction, Eye gaze, Montezuma’s revenge, Sub-goal extraction

## Abstract

While deep reinforcement learning (RL) agents outperform humans on an increasing number of tasks, training them requires data equivalent to decades of human gameplay. Recent hierarchical RL methods have increased sample efficiency by incorporating information inherent to the structure of the decision problem but at the cost of having to discover or use human-annotated sub-goals that guide the learning process. We show that intentions of human players, i.e. the precursor of goal-oriented decisions, can be robustly predicted from eye gaze even for the long-horizon sparse rewards task of Montezuma’s Revenge–one of the most challenging RL tasks in the Atari2600 game suite. We propose *Int-HRL*: Hierarchical RL with intention-based sub-goals that are inferred from human eye gaze. Our novel sub-goal extraction pipeline is fully automatic and replaces the need for manual sub-goal annotation by human experts. Our evaluations show that replacing hand-crafted sub-goals with automatically extracted intentions leads to an HRL agent that is significantly more sample efficient than previous methods.

## Introduction

Recent advances in artificial intelligence (AI) in general, and reinforcement learning (RL) in particular, have shown promising results in developing agents that can interact in complex environments and solve challenging real-world tasks, such as robotic manipulation at scale [1]. Despite these promising results, a key limitation of RL agents is that training them requires extensive exploration and training data. A large body of research [2–6] has thus relied on computer games and other simulated environments to develop and evaluate novel AI agents. One of the most popular testbeds is games from the Atari2600 suite implemented in the Arcade-Learning-Environment (ALE) [6]. The Atari2600 games are particularly useful to evaluate RL agents [7] as they not only have complex visuals but are also challenging for human players [8].

Research on the Atari2600 benchmark has focused on deep RL [3, 4, 9]. While deep RL agents, such as Agent57 [3], have successfully beaten the human benchmark on all 57 Atari games, they are *sample inefficient* and, therefore, require an excessive amount of training. Moreover, deep RL methods suffer from a lack of explainability inherent to the deep neural networks used for Q-value estimation. A more promising approach is hierarchical RL (HRL) [10–12] that decomposes an RL problem into multiple sub-problems, giving structure and improving explainability. A key challenge with HRL is the decomposition of the task that often requires *manual and expert annotations*, which is tedious, time-consuming, and does not easily generalise to other tasks or games.

To address these limitations, we propose a novel approach to automatically identify sub-goals in HRL from human eye gaze behaviour. Eye gaze is particularly promising as the gaze location has been linked to human intentions and goals [13–17]. We hypothesise that these intentions and goals can be further linked to sub-goals so, by predicting players’ intentions from their gaze, sub-goals can be identified automatically. Inspired by prior work on gaze-based intention prediction [13, 15, 16], we extract four gaze features and train a Support Vector Machine (SVM) model. We evaluate the SVM on Montezuma’s Revenge (MR), a long-horizon sparse reward game from the Atari2600 benchmark, with data from Atari-HEAD [7], a data set that offers gaze data in addition to human gameplay demonstrations. Our intention prediction model achieves an average accuracy of 75%, demonstrating the relation between intention and gaze behaviour, which motivates the automatic extraction of sub-goals for hierarchical reinforcement learning (HRL) agents. Finding useful sub-goals, which is also known as the *option discovery problem* [18], is a major issue in HRL. However, by using user intents and gameplay demonstrations, our method is able to not only refine and extract the sub-goals, but also the sequence in which these have to be solved to complete the game level. We then integrate the predicted sub-goals into the HRL framework hg-DAgger/Q [12] and show that this approach can solve the first room of MR three times more efficiently – improving sample efficiency from around 2.3 million to only around 450,000 training steps.

While this strong improvement in sample efficiency also holds for other imitation learning techniques [19] and previous hierarchical methods [11], it is practically limited to environments that are sufficiently static for generating meaningful visual saliency maps. We discuss this limitation in detail and showcase two additional Atari games that fulfill the requirement, namely *Hero* and *Venture*. Both games are similar to MR in that the agent has to navigate different rooms with static layouts and both are also considered hard-exploration games because of their long planning horizon [20]. However, a key difference to MR is the extended amount of possible actions, where the agent can shoot bullets on sprites (Venture) and even place dynamite (Hero). Additionally, they differ in appearance and dynamics of sprites, which makes them an ideal test-bed for our method’s generalisation capabilities. We can show that our automatic pipeline yields meaningful sub-goals for both games, even though they exhibit a very different gaze distribution in the demonstration data set.

In summary, our work makes three distinct contributions: (1) We propose a novel method to predict sub-goals for HRL from eye gaze and human demonstration data. Gaze information is used to predict user intentions that are linked to the sub-goal locations, while demonstration data provides the order in which these sub-goals have to be solved to complete a task. (2) We evaluate our approach on MR from the Atari2600 benchmark and demonstrate significant improvements on two key limitations: sample efficiency and the need for manual expert annotations. (3) We extend our approach to two additional Atari games and show its generalisation capability to other navigation games. The pipeline requires minimal amount of demonstration and gaze data and is therefore suited to be easily integrated with other HRL agents, effectively solving the option discovery problem. Our results pave the way towards new intention-based HRL methods that leverage both hierarchical methods and additional human behavioural data, such as eye gaze, to train more efficient agents that can solve complex environments.

## Related work

Deep reinforcement learning (RL) has shown great results on the Atari benchmark but still struggles to learn robust value functions from sparse feedback in long-horizon games, such as Montezuma’s Revenge (MR). While many solutions have been proposed [3, 4, 21], they require frame samples in the range of billions, which forces researchers to develop elaborate distributed training schemes that still take a considerable amount of time to train [2]. Among others, transfer learning has been proposed, such as inter-agent transfer learning [22, 23] that leverages instructions from an expert agent for faster convergence. However, the domain shift between the training domain of the expert and the task domain still poses a significant challenge. Hierarchical reinforcement learning (HRL), on the other hand, offers a way of exploiting the hierarchical structure of decision-making tasks, guiding the agent towards meaningful sub-goals, and effectively increasing the sample efficiency of agents. Moreover, agents achieving consecutive sub-goals, are directly interpretable, making HRL particularly useful in domains, where explainability is required.

### Hierarchical reinforcement learning

Early on, even before deep RL, two ideas have emerged in HRL: the options framework [24] and feudal networks [25]. Sutton et al. [24] have proposed to temporally extend actions into *options*, which are composed of a policy, a termination condition, and a set of states in which they could be applied [24]. They have shown that Q-learning could be generalised to learning policies over options and that learning inside these options, called "intra-option" learning, allowed the agent to learn about the respective options without executing them explicitly. Feudal networks, on the other hand, define a hierarchical structure of managers and sub-managers that are only privy to the space and temporal state at their granularity, effectively hiding information from their superior and providing rewards to their sub-managers even if their superior goal was not satisfied [25]. Both hierarchical frameworks have demonstrated much faster convergence than non-hierarchical methods in their respective maze scenarios.

More recently, Kulkarni et al. have proposed a hierarchical approach to induce goal-directed behaviour that does not use separate Q-functions as in the options framework [11]. This made their method scalable and promoted shared learning between options. To this end, they proposed a two-level framework in which the top-level agent (meta-controller) was responsible for choosing sub-goals, while the low-level agent was concerned with achieving these goals. Le et al. have extended this approach by integrating the interactive imitation learning approach DAgger [26] into the meta-controller [12]. This, however, introduced the need for an expert during training. Their approach is also similar to feudal networks [10, 25] in their hierarchical structure; however, it needs significantly less data as it does not use standard RL on the higher level. Vezhnevets et al. have later argued that a disadvantage of [11] is the need for pre-defined sub-goals and have chosen to learn goal embeddings implicitly [10]. Subsequently, Veeriah et al. [27] have proposed to learn the sub-goals directly in the RL framework, using additional networks for generating and evaluating new sub-goals, as well as primitive actions based on past observations and a task encoding. While this approach can be trained without any external data, it significantly increases the complexity of the learned model. Due to the training of the additional networks, the efficiency gains of the hierarchical approach are annihilated, as demonstrated in [27]. In this work, we take the best of both worlds and leverage the information provided by gaze data to extract sub-goals independently. This allows us to train sample efficiently based on hierarchical guidance, while neither requiring human supervision for sub-goal selection, nor additional networks for sub-goal prediction.

Another work developed concurrently with ours is based on the options framework but also defines intentions as fully satisfied if a sub-goal is reached and evaluates a reduction in available actions to the ones that are affordable in a given state (affordances) via attention. Nica et al. [28] introduce these *affordance-aware sub-goal options* with a respective model-free RL algorithm and find empirically in a MiniGrid domain that this yields better sample efficiency and higher performance in long-horizon tasks. While they also incorporate visual attention, they do so by applying it to limit an agent’s action choices. In our work, on the other hand, we use visual attention maps generated from eye gaze data to extract meaningful sub-goals that can be directly selected by the meta-controller of our more feudal network-like architecture.

### Intention prediction

Intentions are goals and desires associated with a concrete plan, i.e. an intention causes a sequence of actions that lead to achieving a certain goal [29]. In other words, intentions are the precursor of actions, which poses the question of whether human intentions are able to pose as sub-goals for HRL agents, where the hardest problem is to discover suitable sub-goals [18]. However, human intention prediction has never been done in this context before. Therefore, before we can replace hand-crafted sub-goals with human intention, we verify whether intention prediction is feasible on MR.

Model-free intention prediction models rely on eye gaze as the most important feature [13–17]. For a tabular summary of intention and activity recognition using eye gaze in Virtual Reality (VR), PC, table-top, and real-world environments, we refer the reader to Chen and Hou [16]. The work of Huang et al. [13] is the most relevant to ours, as they consider intention prediction as a multi-class classification in a real-world scenario. They achieved 89% accuracy in their collaborative ingredient prediction task, where a customer instructs a worker to add displayed ingredients to a sandwich, and 76% accuracy with gaze features alone. The gaze features used in their Support Vector Machine (SVM) model were: *total duration of looks*, *most recently looked at*, *number of glances*, *duration of first glance*. We successfully test their model on MR with gaze data from the demonstration data set Atari-HEAD [7].

Belardinelli [17] offers a more general review on gaze-based intention estimation, identifying application areas of intention prediction as human-computer interfaces, human-robot interaction, and Advanced Driving Assistance Systems (ADAS) with relevant works from the last decade of research. However, the application of intentions to solve the *option discovery problem* in HRL, or in our case, the *sub-goal discovery problem*, is, to the best of our knowledge, a novel idea and constitutes the main contribution of our work.

## Sub-goal discovery

**Fig. 1:**
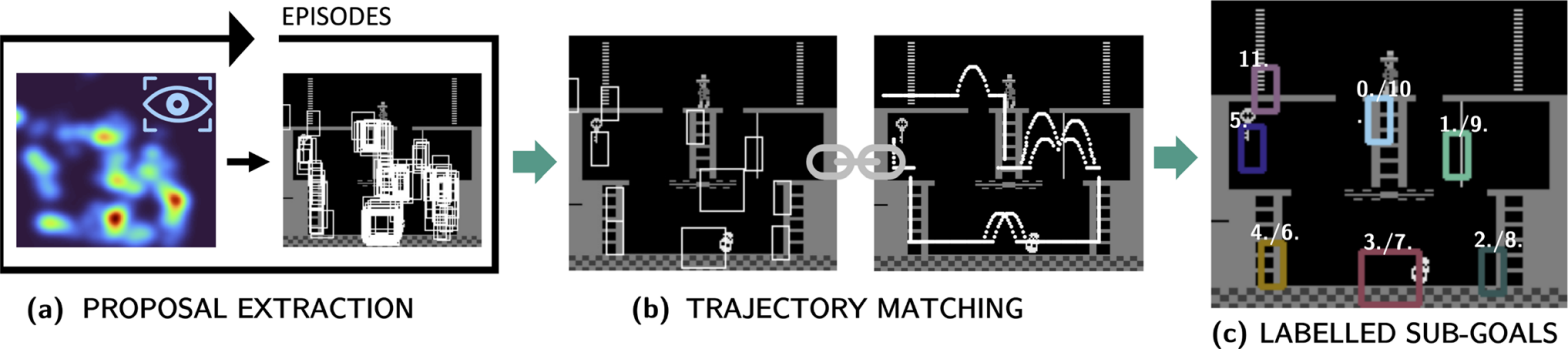
Sub-goal extraction pipeline: **(a)** proposal extraction is performed from human attention maps for each episode and resulting proposals are merged via non-maximum suppression (NMS), then final proposals for one room are matched with human agents’ trajectories **(b)**, yielding labelled sub-goals and visitation order **(c)**

MR is one of the most challenging games in the Atari2600 suite because of its long planning horizon and sparse rewards [12]. Unlike similar long-horizon planning tasks, e.g. artificial grids [18, 28], MR is more challenging because it features different rooms that change according to the current level and collecting items allows for different actions in them. Therefore, to identify a specific state of the game, it is necessary to know the position of the agent, room ID, level number, and the number of keys held [30]. The room ID is particularly important for our method because gaze data should be evaluated separately for each room so that gaze points can be mapped to the specific areas of interest.

The required state information can be extracted directly from the Arcade-Learning-Environment (ALE) via an environment wrapper called Atari Annotated RAM Interface (AtariARI) [31]. The wrapper parses information from the state variables in the ALE and makes it available for each environment step. However, the AtariARI wrapper was not used in the collection process of the data set Atari-HEAD [7]. To acquire the necessary labels subsequently, we simulated the episodes played by humans. This was possible as the original collection was done in a frame-by-frame mode, labelling each consecutive action.

### Sub-goal extraction

Our method for sub-goal extraction is inspired by previous research that showed that visual attention is a predictor of human intentions [13, 15–17, 32] and is further validated by successfully performing intention prediction on the extracted sub-goals in room one. The novel extraction pipeline is visualised in Fig. 1: separate visual saliency maps are calculated for each episode and further isolated to only include the gaze data from the first room in the first level. These saliency maps are generated by adding each gaze point to the frame and passing a Gaussian filter over the generated fixation map with variance $\sigma$ being one visual degree (pixel / visual degrees of the screen). Finally, the saliency map is normalised into the range of [0, 1]. An example saliency map can be seen in Fig. 1 (a), where hot areas (red/yellow) indicate a high focus of attention across the selected time frame, and cold areas (blue) were not gazed upon at all.

**Fig. 2:**
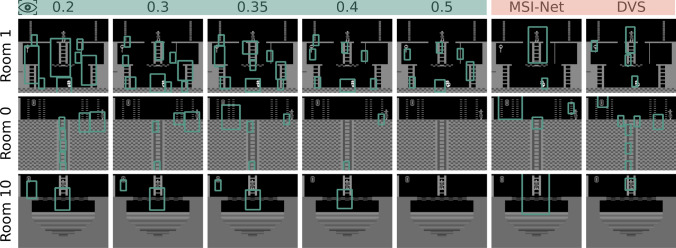
Sub-goal extraction examples on three rooms of MR with different saliency map thresholds from 0.2 to 0.5 on human gaze data, as well as with saliency maps generated by the MSI-Net [34] and DVS [35] saliency models with a fixed threshold of 0.4

After generating saliency maps for all episodes, each saliency map was thresholded, i.e. only values above 0.4 were kept. This threshold was fine-tuned to yield the best results over the entire sub-goal extraction pipeline; a qualitative assessment of other thresholds can be seen in Fig. 2. If the threshold is set too low (e.g. here 0.2), potential sub-goal bounding boxes are more frequent and as in room one span almost the full area. If the threshold is too high (e.g. here 0.5), on the other hand, there are not enough sub-goal proposals (room one), or none at all, as seen in room zero and room ten. With the fine-tuned threshold, sub-goal proposals were generated, by drawing an agent-sized bounding box around each remaining saliency map point. These proposals were then processed with a custom implementation of the non-maximum suppression algorithm (NMS) [33]. In general, NMS is applied to suppress overlapping bounding boxes if they exceed an Intersection-over-Union (IoU) threshold, and, in our case, boxes with higher saliency values were favoured. Then, the remaining overlapping boxes were merged into one. After the number of sub-goal proposals for each episode was greatly reduced in this manner, the process was repeated to combine proposals across all episodes, yielding a final number of 11 possible sub-goals for room one, as shown in Fig. 1 (b).

With the definition of intention in mind, where intention directly leads to goal-directed behaviour [29], it is intuitive to only include sub-goals as labels for intentions that are visited during gameplay. Therefore, we ran another simulation of the game data from human players to find the sub-goals that were visited and in which order (trajectory matching). As MR is considered to be an almost deterministic game, i.e. there exists a best sequence of sub-goals, this resulted in almost identical orders across episodes. The remaining discrepancies were rectified by implementing a majority vote.

Overall, this extraction procedure resulted in 7 remaining sub-goals, labelled in order as shown in Fig. 1 (c): moving from the middle ladder (0) to rope (1) and bottom-right ladder (2), to crossing the middle area with the dangerous and dynamic skull (3), to the bottom left ladder (4), climbing up to collect the key (5), and then reversing this order to get to the left door (11). Interestingly, in all the episodes collected of human gameplay, only the left door was used, most likely because this is the best route suggested in MR solution guides.

In comparison with the four hand-crafted sub-goals selected by the HRL framework of Le et al. [12], which they hand-picked from the six sub-goals manually selected by Kulkarni et al. [11], our automatic pipeline extracted the same goals and added more areas of interest. In detail, Kulkarni et al. originally performed object detection on the image of room one and then chose the two doors, the three ladders, and the key as entities to define relational goals in the form of *agent* *reaches* *goal*.

### Sub-goal analysis

We further analysed our sub-goal extraction pipeline (Fig. 1) qualitatively by generating proposals and final sub-goals for additional rooms of MR, with different saliency map thresholds, but also with artificial saliency maps generated by saliency models [34, 35], the results of which can be seen in Fig. 2. We showcase *Room 1* as the starting point of the game, *Room 0* as the second room reached when choosing the left door, and *Room 10* as it features a special room layout. The saliency map threshold is a hyperparameter that needs to be fine-tuned on the overall extraction, where we have chosen 0.4 as it includes all hand-crafted sub-goals proposed by prior work [11, 12] with the meaningful addition of the area around the skull, without adding insignificant goals as with lower thresholds, but still including the door, which would be left out by a higher threshold. While there are no hand-crafted sub-goals to compare to for other rooms of MR, we can see that the pipeline also selects meaningful sub-goals, e.g. the bottom pathway in *Room 0*, the disappearing floor in *Room 10*, or the diamonds that give an external reward in both. In contrast, artificially generated saliency maps by MSI-Net [34], a standard saliency model with state-of-the-art results on the saliency benchmark CAT2000 [36], and DVS [35], a saliency model optimised for data visualisations, have a predominant focus on the agent itself and otherwise fail to find important steps like the doors, even though they are highlighted in the same colour as the key. Note here that saliency map prediction was done on the RGB images.

For testing the intention prediction model of Huang et al. [13] on our extracted sub-goals, we preprocessed the gaze data following prior work [15, 16], extracting saccade and fixation events and calculating the four features: *total duration of looks*, *most recently looked at*, *number of glances*, *duration of first glance*. We then implemented intention prediction as a multi-class classification for the 7 sub-goals of room one with a SVM. In detail, we fine-tuned a standard SVM classifier with RBF kernel via grid-search, yielding a L2 regularisation value of $C=510$. This intention predictor reached an average accuracy of 72.8% (std 5.5%) in a 10-fold cross-validation on our data set of 20 episodes from the Atari-HEAD [7] demonstration data, significantly outperforming the baseline of random chance with $\frac{1}{7} = 14.3\%$. We argue that this corroborates the efficacy of using human gaze data as an indicator of intention and motivates the extraction of sub-goals for HRL from human intention.

## Intention-based learning

### Baseline

One approach for solving long-horizon decision-making tasks is HRL, where two popular frameworks emerged in the past: the options framework [24, 28] and feudal networks [10, 25]. Building upon a feudal architecture, by combining deep HRL with pre-defined sub-goals, Kulkarni et al. [11] are able to outperform naïve deep Q-learning. Their h-DQN model was tested on two delayed-reward domains, including the first room of MR, where their approach is able to reach the door after 2.5 M samples. Taking the idea further, Le et al. [12] combined imitation learning (IL) with HRL showing that their hierarchical guidance model (hg-DAgger/Q) significantly reduces expert effort compared to other interactive IL approaches and is also able to learn faster and more robustly than h-DQN, solving the first room of MR after 2.3 M steps.

Our baseline, the hg-DAgger/Q model by Le et al., consists of two levels, the meta-controller level, and the agents level. They use the data aggregation method DAgger [26] on the top level, which trains the meta-controller policy with iteratively aggregated data sets. The meta-controller is used to predict one of the four hand-crafted sub-goals, initiating the corresponding agent.

On the low level, Le et al. used a double deep Q-Network (DDQN) [37] with prioritised experience replay [38]. A major difference between their approach and h-DQN [11] is that separate agents are trained for each sub-goal instead of passing the goal vector as a feature into a single policy network. This ensures the mitigation of the issue of catastrophic forgetting and also has the advantage of separate exploration schedules. However, maintaining a separate network for each sub-goal is not scalable across different rooms of MR.

Combining the low-level RL agents with hierarchical guidance from the meta-controller ensures that the experience buffer for the DDQN only contains valuable samples for the next sub-goal, as wrong meta-controller choices terminate the episode. Le et al. argue that this is the main reason for their higher robustness in training. However, Le et al. also report that their architecture only learned all sub-goals successfully in 50 out of 100 trials. This high variability is most likely due to different implementations and random seeding, an issue common in RL [39], which would also explain, why we were unable to reproduce their results. Consequently, we will compare our results to the ones reported in their paper.

In summary, hg-DAgger/Q [12] is significantly more sample efficient than other methods [10, 11]. However, it requires an expert at training time to select hand-crafted sub-goals and only implements a rudimentary sub-goal check.

### Int-HRL model

Similar to [12], we use a hierarchical reinforcement learning approach, with 8 possible actions (no action, cardinal moving directions, jumping up, left, and right). We keep training hyperparamaters consistent with hg-DAgger/Q, i.e. an experience replay buffer of size 500,000, frame skip and action repeat of four for the environment, a linear scheduler for epsilon greedy exploration (from 1.0 to 0.02 in 200,000 steps), a network with three convolution layers, ReLU activation and batch normalisation, followed by two linear layers starting with 512 hidden nodes, trained with an Adam optimiser with learning rate $1\text {e-}4$. While we ran multiple experiments with different hyperparameters, we found only the following adaptions to consistently improve training stability: First, by using a dueling deep Q-Network (DQN) [40] architecture in addition to the DDQN [37]. Second, only by including the lower left ladder as an additional hand-crafted goal, we were able to train the first hg-DAgger/Q agent to reach the first external reward, the key.

While Kulkarni et al. [11] used six different sub-goals, namely the two doors, the three ladders, and the key, Le et al. [12] only chose the right door and the bottom-right ladder, which they count twice, in addition to the key. For our method, we replace these hand-crafted sub-goals with the fields of interest derived from human gaze data, as previously described, thereby expanding the set of sub-goals used in [11, 12] to the 7 unique goals shown in Fig. 1 (c). Interestingly, our human gaze analysis indicates that all six goals used by [11] are relevant, even more so, that the rope and the dangerous area around the skull are additional fields of interest.

In addition to their importance for HRL, sub-goals are a way of providing pseudo rewards [11, 12, 24] to populate the sparse reward map in MR. Next to the sub-goal reached reward $R_{\text {sub-goal}}$, we introduce a dense reward signal $R_{\text {dir}}$, $R_{\text {dist}}$, and $R_{\text {step}}$ to further stabilise training.

1 $$\begin{aligned} R = R_{\text {sub-goal}} + \alpha R_{\text {dir}} + \beta R_{\text {dist}} + \gamma R_{\text {step}} \end{aligned}$$

We define a direction reward $R_{\text {dir}}$ to steer the agent in the direction of the next sub-goal. It is computed as the scalar product of the selected action’s direction vector $\vec {a}$ and the vector between the next and previous goal $\vec {g} = G_{prev} - G_{next}$:

2 $$\begin{aligned} R_{\text {dir}}&= <\vec {a}, \vec {g}> \end{aligned}$$

The distance reward $R_{\text {dist}}$ guides the agent to the next sub-goal by minimising the Euclidean distance between the agent and current goal $d_{ac}$, as well as the previous goal $d_{ap}$, and the distance between previous and current goal $d_{pc}$:

3 $$\begin{aligned} R_{\text {dist}}&= \frac{\sqrt{d_{ap}} - \sqrt{d_{ac}}}{\sqrt{d_{pc}}} \end{aligned}$$

The step reward $R_{\text {step}}$ penalises each time-step used to reach the next goal with a constant $\tau = 0.001$, which is also used to scale $R_{\text {dist}}$ and $R_{\text {dir}}$. In general, we represent the sub-goals with their respective bounding box coordinates, however, for all distance and direction calculations we use the centre of the respective sub-goal’s bounding box.

While the direction reward $R_{\text {dir}}$ is simple to implement, the distance reward $R_\text {dist}$ requires the agent location at each step. Here, we propose to either use a specifically trained object detector, which we tested with a pre-trained FasterRCNN model [41] fine-tuned on 100 manually labelled training examples or to use the RAM state labels provided via the AtariARI Wrapper [31]. Both of these approaches are more robust than the approach used in [12] and, additionally, are able to track other non-static regions of interest.

**Fig. 3:**
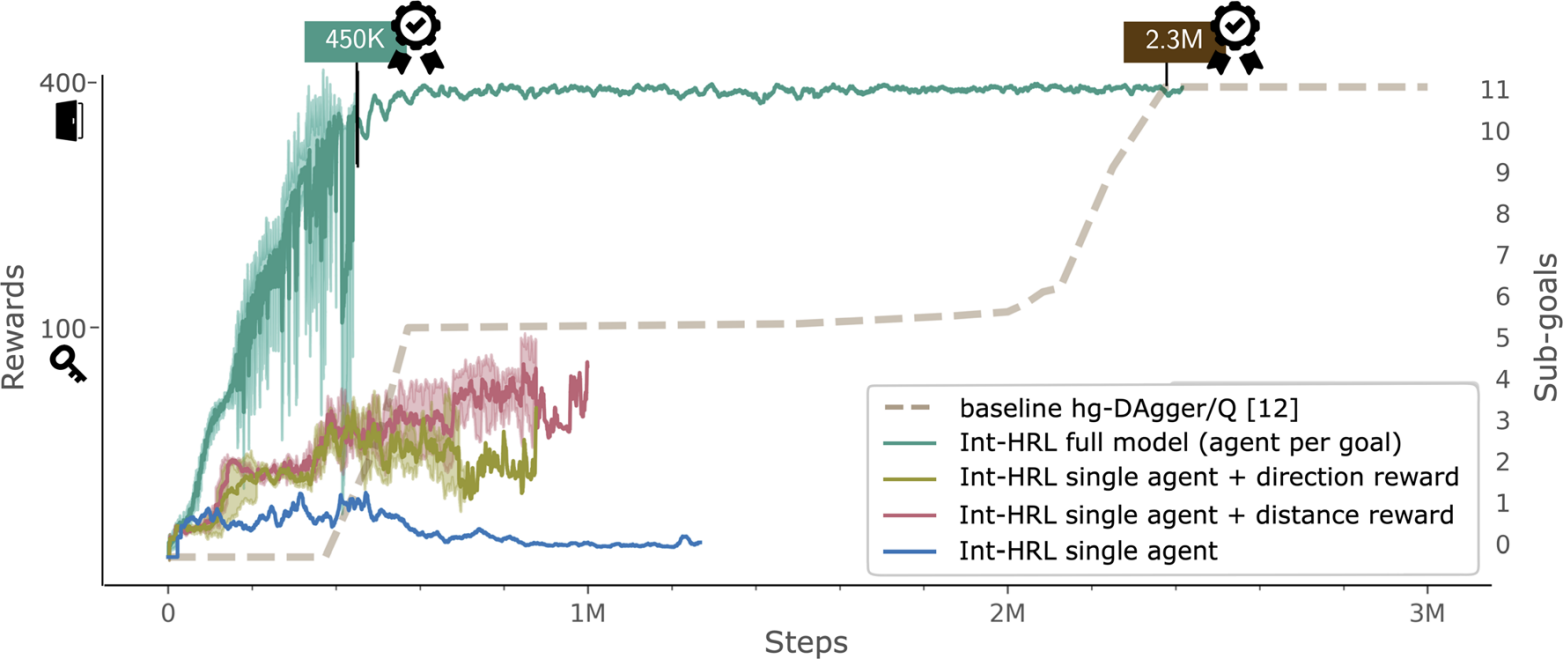
Step-wise sub-goal trailing performance of Int-HRL and baseline with sub-goal 5 as a first external reward from the key and sub-goal 11 the right/left door, which completes the first room

## Results

We evaluated the full-sequence hierarchical model without any dense reward, a single-agent model with negative step reward and goal feature: $\gamma = 1$, and the single-agent model with a distance reward: $\beta = 1$ in comparison with the direction reward: $\alpha = 1$. To further help the single-agent models, we passed the sub-goals as additional feature vectors, i.e. we add the four bounding box coordinates of the respective sub-goal to the 512 hidden nodes before they get passed into the two linear layers. Throughout all experiments, we used an $\epsilon$-greedy exploration policy with a linear scheduler ranging from 1 to 0.02 in 200 K steps, a prioritised replay buffer [38], and a learning rate of 0.0001. The results of this experiment are shown in Fig. 3. In line with previous work, we show reward and sub-goals together for easier comparison with the baselines [11, 12]. Each sub-goal is considered learned when the trailing performance is above 0.9, i.e. when the agent reaches the goal in 90 out of 100 trials. Directly compared models are trained with the same random seed to ensure comparability [39].

The full model is the first to successfully learn to solve the first room of MR by reaching the left door. The final goal (11) is consistently reached by the full model after only 450K steps. Hence, our model is more than four times more sample efficient than our baseline [12], which needs 2.3M samples to complete the first room. Furthermore, our new framework works without an expert, as we extracted the chosen sub-goals previously from gaze data and perform a true goal reached check via RAM state mapping. Overall, given labelled gaze demonstration data, the pipeline can be trained end-to-end with no manual effort.

In comparison with the full model, the single-agent model with the goal feature and small negative rewards per step does not succeed at all. As expected, the single dueling DDQN agent suffers from a lack of *exploration* [12], i.e. as Fig. 3 shows, the single-agent model fails to learn anything new and gets stuck at the first sub-goal. Resetting the schedule when a new sub-goal is explored was tested, but did not succeed as it resulted in $\epsilon$ being too high, which also prevents learning. Further ideas for solving insufficient exploration are included in the discussion and are left for future work.

Providing a more dense reward structure by adding a distance or direction reward improves the single-agent model as it increases sample efficiency so that the model learns to reach sub-goal 4, i.e. passing the skull and reaching the lower left ladder (see the distance and direction reward model in Fig. 3). In our trials, the distance reward even facilitates learning to reach the key sub-goal (5), which provides the first external reward. While performing slightly worse, the direction reward is simpler to implement as it does not require knowledge of the agent’s location. Both models still suffer from the issues encountered by the single agent, and performance deteriorates after 200K steps when the $\epsilon$-exploration schedule reaches its final value. However, this confirms that more intrinsic rewards improve performance and should, therefore, be incorporated into future models.

### Generalisation

To evaluate the generalisability of Int-HRL, we evaluated it on two other Atari2600 games, namely *Venture* and *Hero*. Both games are considered hard-exploration games and cannot be solved with standard deep learning approaches [20]. We have selected them because they share important similarities with MR, as they are navigation games with different rooms and levels, where items need to be collected (Venture) or miners need to be rescued (Hero), while sprites and other obstacles need to be overcome. The separate rooms allow for a natural partitioning of gaze data and static items are good candidates for sub-goals. These characteristics make both games suitable for our method. While both games require the learning of more actions, i.e. 18 instead of 8 as in MR, they also have a more dense reward structure. Additional differences to MR are in their appearance and the mobility of dangerous sprites. The successful application of our method, therefore, showcases the generalisability to other navigation environments with clear gaze partitions.

The pipeline is the same as for MR: First, we label the demonstration data from Atari-HEAD [7] to include room and level ID, as well as player positions, with the AtariARI wrapper [31]. Then, we generate saliency maps from gaze data for each room and episode separately and use a threshold to turn them into bounding box proposals (see Fig. 1 a). These bounding boxes are then merged and decimated via NMS and the resulting sub-goal proposals are further refined by trajectory matching, i.e. by checking whether the players visit them (see Fig. 1 b).

**Fig. 4:**
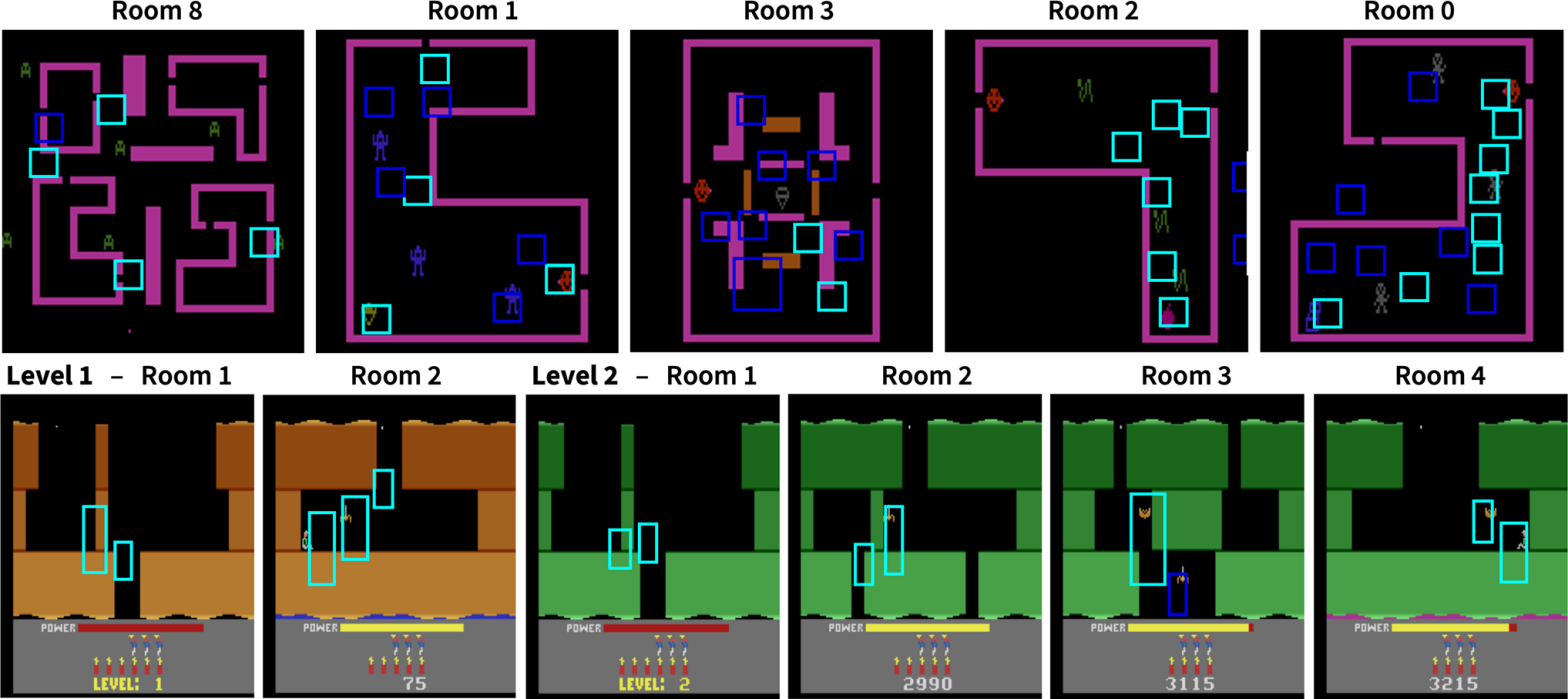
Sub-goal proposals (blue) and final annotations (cyan) for Atari game *Venture* (top) and *Hero* (bottom). For both games our pipeline yields meaningful sub-goals, corresponding to external reward locations (e.g. items), but also important areas that need to be navigated and dangers to be evaded

#### Venture

Venture is an action game in which the player needs to explore different rooms and collect treasures, while evading and shooting sprites. It is considered one of the 10 most challenging Atari games [3], as it requires long-time planning. The player starts in an overview room (see Fig. 4 room 8), from which four other rooms can be reached. To complete this first level, an item has to be collected in each room without running into sprites or movable walls, e.g. in room 3. Once all items have been collected and the last room has been left, the level concludes automatically and the player starts in a new overview room. As can be seen in Fig. 4, Venture is not as deterministic as MR and it also uses the full action map with 18 possible actions, since the additional firing action can be combined with all movement actions. Another key difference is the smoothness of the visualisations; in Venture, the frames flicker constantly, as many objects are only rendered every second frame; most likely, this was due to the capacity limits of the original Atari console. Moreover, the sprites that need to be evaded or shot are more dynamic and move across the entire rooms in contrast to MR, where the only sprite, the skull, is confined to a small area on the bottom.

Our extraction pipeline yields more sub-goal proposals (see Fig. 4: top row blue) for Venture than for MR, even with an updated saliency threshold of 0.9. Therefore, the trajectory matching step becomes more important. However, as can be seen for room 2 and room 0, the trajectory matching still results in more final sub-goals. The higher amount of sub-goals makes running a full model with separate agents for each sub-goal, as in [12], computationally infeasible. On the other hand, it provides an even denser reward structure and facilitates the learning of our single agents with goal feature $R_{\text {sub-goal}}$ and distance reward $R_{\text {dist}}$. While the simple sub-goal feature agent is not able to complete the first room because it wanders around too much and gets killed by sprites, the single agent with distance reward solves the room after 560K steps. In comparison with MR, where the first room with 12 sub-goals is solved after 450K steps it is much slower, however, in MR the only model capable of successfully solving the first room is the full agent model. Here, the single-agent model requires significantly less resources. We cannot provide further baseline results as [11, 12] have not been evaluated on any games except MR and both would require expert annotation of sub-goals.

Analysing the gaze distribution for Venture, we find that gaze on the agent ($21\%$) and its vicinity ($47\%$) is lower than in MR with $33\%$ and $68\%$, respectively. Additionally, the percentage of gaze on sub-goals ($31\%$) is also significantly lower than for MR ($57\%$). While this can be partly explained by the larger agent size in MR and the higher amount of sub-goals in Venture, we hypothesise that attention bias plays another key role. The flickering of frames due to partial rendering, which specifically affects the enemy sprites, attracts visual attention [42]. Furthermore, the number of sprites and their dynamics is much higher in Venture, therefore, visual attention is less focused on single areas and more dispersed across the entire screen. In summary, guiding the agent with constant feedback towards meaningful sub-goals is particularly beneficial in Venture, as agents with less supervision get easily distracted by the characteristics of the game.

#### Hero

In Hero, the player also navigates different rooms and has to evade sprites but the overall goal is to rescue miners lost in tunnels and the game iteratively expands the number of rooms for each level. For example, in Fig. 4 the first two levels of Hero are depicted; while the first room stays the same, level 2 becomes deeper with additional rooms up to the final room 4. A key characteristic of this game is the player’s ability to place dynamite to tear down walls, which is necessary, e.g. in room 1 of both levels, to reach the tunnel to go further down. Additionally, as can be seen in Fig. 4, almost half of the screen is dedicated to a display of game statistics, such as the power bar, number of lives as player icons, and number of remaining dynamite. The power is required for the jet pack to smoothly fly down the tunnels, if it is empty, the player falls down and looses a life. Therefore, player’s attention is drawn to the power display, in fact, in the demonstration data from Atari-HEAD [7], $39.9\%$ of the time the player’s gaze is on the display. Hero is particularly interesting, as it is considered challenging [20] and because of the distinct skills that an agent needs to master, e.g. placing dynamite, while maintaining attention on the available resources.

To facilitate more meaningful sub-goal proposals from the start, we have excluded gaze data that was below the player screen, i.e. on the display, as it would not yield reachable sub-goals. However, there is a clear attention bias towards the bottom of the screen and, therefore, sub-goals for Hero tend to have a small y-axis offset. Apart from the y-axis offset, our sub-goal extraction pipeline yields ideal sub-goals, where each corresponds to either an external reward location, i.e. rescuing the miner or shooting sprites, or important navigation areas, where dynamite has to be placed or tunnels need to be explored. Interestingly, the gaze distribution on the Hero agent is significantly lower than in Venture or MR with only $13.4\%$ and $29.5\%$ on its vicinity, most likely due to the aforementioned attention bias towards the player statistics display. However, the percentage of gaze on the extracted sub-goals remains similar to Venture with around $33.7\%$. In contrast to Venture, where there are a lot of sub-goal proposals, in Hero all proposals but one are matched with the agent’s trajectory. In detail, in room 3 of level 2, the sub-goal proposal is on one of the sprites, but because the agent can simply fly past the enemy, the trajectory matching algorithm excludes this proposal. This means, for Hero, the final trajectory matching step is mainly used to determine the sub-goal order. In this regard, Hero is similar to MR with a deterministic order of tasks that need to be performed.

Our single model with distance reward is able to solve the first room of Hero after only 120K steps, which includes acquiring the skill of placing dynamite and moving out of the way. Surprisingly, the single model works better than the full model here, most likely because the full model starts a new agent after reaching the first sub-goal, which then begins to explore from scratch and often gets killed by placing dynamite. However, as mentioned before, the single model is preferred over the full model, as it requires much less resources and could be scaled for solving the entire game.

## Discussion and outlook

We have shown that gaze features are indicative of intentions by successfully training a simple intention prediction model on the first room of the Atari game MR. Further, we developed a novel sub-goal extraction pipeline from gaze data. To this end, we labelled an available demonstration data set via simulation, analysed the visual attention heatmaps for each room, and aligned the proposals with the agent trajectories. This process yields sub-goals on MR that are on par with hand-crafted ones from prior work [11, 12] and objectively meaningful sub-goals on games, where no comparable expert annotations exists. We demonstrate the efficacy of our sub-goal extraction pipeline by using the extracted sub-goals to train an HRL agent that can solve the first room of MR significantly more sample efficiently than any previous method. Moreover, our pipeline is fully automatic and allows for a transparent explanation of agent behaviour. In comparison with previous methods, where sub-goals have been chosen manually without further analysis [11, 12].

**Generalisability to other Atari games.** We have chosen the long-horizon sparse reward game MR of the Atari2600 suite because it can be structured into sub-tasks across different static rooms and standard RL still struggles with solving it efficiently. Other games available in the Atari-HEAD [7] data set are not suitable for this analysis because agents and sprites can move across all lanes, because objects of interest scroll too fast across the game screen, or because areas of interest are trivial, as in shooter games, where all sprites are at the top of the screen and agent movement is restricted to be horizontal across the bottom.

Other games similar to MR are *Hero* and *Venture*, where different rooms need to be navigated, which include static sprites or objects and clear goals. While *Hero* is structured like a search tree, iteratively expanding the depth of exploration for solving a level by finding people lost in the caverns, *Venture* is like a Maze with an overview screen from which the agent can reach different rooms to find treasure. We have shown the generalisability of our method on these games by running our sub-goal extraction pipeline, where only the threshold parameter needs to be tuned. While both games exhibit a unique gaze distribution with attention bias towards flickers (Venture) or a resource display (Hero), our gaze-based extraction method still found meaningful sub-goals, either corresponding to external rewards or areas that are difficult to navigate.

**Generalisability to other domains.** One requirement for our approach is a fixed layout of rooms for extracting meaningful information from gaze data. Areas of interest need to be stationary enough for a high duration of attention and depict isolated or unique objects. This limits the approach to specific task domains, a key limitation that is not present for other imitation learning methods [12, 19]. While our method is unlikely to succeed in 3D or first-person view domains like VizDoom [43], there are other potential application domains [44–46]. For example, Mannering et al. [47] have shown the efficacy of predicting intentions for the collaborative multi-agent game Overcooked, a test-bed for zero-shot cooperation that might be tackled with intention prediction and Theory of Mind [44]. Additionally, navigation environments as found in POPGym [45], specifically labyrinth escape and explore, or the more complex tasks of MiniGrid [48], or MiniGrid’s extension Xland-MiniGrid [46] might benefit from intention-based HRL.

**Demonstration and gaze data requirements.** In contrast to other gaze-assisted approaches [19, 49] using IL, we do not require demonstration data during training. Nor do we need expert annotations as in our baseline [12]. In fact, our method only requires data from one naive demonstrator, as proven with MR, where there was only a single annotator in our demonstration data set Atari-HEAD [7]. Therefore, Int-HRL is not only sample efficient in terms of RL steps, it is also sample efficient in terms of demonstration data and does not require the costly collection of a large data set.

**Scalability of HRL method.** While the full-sequence model has outperformed all other baselines tested on the first room of MR in terms of sample efficiency [10–12], it needs to be more scalable to solve the entire game. The HRL approach based on Le et al. [12] requires the separate handling of 12 agents in the first room of MR, but an additional 23 rooms need to be explored to solve the first level. While both [10, 11] only use a single low-level agent, the former’s successful trials were not reproducible [12] and the latter requires 200 M samples for the first room alone, most likely getting close to the 10 billion samples needed by standard deep RL approaches [3]. We have tested single agents with more dense reward structures ($R_\text {step}$, $R_\text {dist}$, $R_\text {dir}$). However, we were unable to circumvent the issue of *insufficient exploration* in single agents. In future work, we would like to address this by adding per-episode and full-game novelty values as intrinsic rewards, which succeeded in deep RL methods [3, 9].

## Data Availability

The data set Atari-HEAD by Zhang et al. [7] used for our analysis is publicly available at https://zenodo.org/records/2587121.
